# Factors influencing junior high school students’ perceptions of attending school in Japan

**DOI:** 10.1186/s13034-023-00631-w

**Published:** 2023-07-04

**Authors:** Hiromi Nakamura-Thomas, Nobuyuki Sano, Donald Maciver

**Affiliations:** 1 Graduate School of Health, Medicine and Welfare, School of Health, Medicine and Welfare, Saitama Prefectural University, 820 San-No-Miya, Koshigaya City, Saitama Japan; 2 Department of Occupational Therapy, Faculty of Medical Sciences, Fukuoka International University of Health and Welfare, 3-6-40 Momochihama, Sawara-Ku, Fukuoka City, , Fukuoka Japan; 3 School of Health Sciences, Division of Occupation Therapy and Art Therapies, Queen Margaret University, Queen Margaret University Way, Musselburgh, EH21 6UU UK

**Keywords:** School attendance, Positive relationships, Friends, School teachers, Junior high school, Parents

## Abstract

**Background:**

School attendance is a crucial determinant of academic success. Our previous research has identified factors that influence elementary school students’ perceptions of attending school, but whether these factors apply to older students remains unclear. We investigated the extent to which the factors identified in the previous research apply to junior high school students and their attitudes toward attending school.

**Methods:**

We hypothesized that students’ “perceptions of attending school” was directly influenced by their perceptions of “relationships with friends and teachers,” “current circumstances,” “subjective health status,” and “having people to share experiences and thoughts with.” We developed an original questionnaire with 19 items and analyzed data collected from 6245 junior high school students in Japan, using a structural equation model.

**Results:**

The final model demonstrated a good fit. Students’ “positive perceptions of attending school” was directly and positively influenced by their “positive perceptions of relationships with friends and teachers” and directly and negatively influenced by their “perceptions of poorer subjective health status”. Other latent variables directly and positively influenced the perceptions of attending school, but not strongly. Students’ perceptions of “relationships with friends and teachers,” “current circumstances,” and “having people to share experiences and thoughts with” correlated positively with each other. These three latent variables also correlated negatively with “poorer subjective health status.”

**Conclusions:**

The role of positive relationships with friends and teachers in shaping students' perceptions of school attendance, coupled with the negative impact of poorer subjective health status, underscores the need for educators to adopt approaches that specifically address these areas. It is crucial to provide support to students in cultivating positive relationships, fostering positive perceptions of school, and offering resources to those who are encountering mental or physical health challenges. Implementing the evidence-based questionnaire developed in this study is recommended to enhance student support and well-being.

**Supplementary Information:**

The online version contains supplementary material available at 10.1186/s13034-023-00631-w.

## Background

School attendance and academic success are fundamental for children and adolescents [1]. School should recognize absenteeism for any reason as a serious warning sign and take steps to remedy the situation [2]. Meta-analytic data show that school absenteeism is a risk factor for critical outcomes, including self-harm and suicidal ideation [3]. Various school-based interventions aimed at preventing and improving school nonattendance have been provided; however, it remains one of the major issues in education in many countries, inducing Japan [4]. Researchers have identified a significant group of students who attend school but are at an elevated risk of school avoidance, as they experience negative emotions towards school that have the potential to develop into more severe issues with avoidance [5]. In Japan, a recent nationwide survey revealed that more than 10% of 6450 current junior high school students did not want to go to school [6].

When students demonstrate reluctance to attend school, exhibit reduced attendance, or completely avoid attending, these actions are often accompanied by the existence of distressed behaviors and anxiety [7–11]. One of the recommendations about how to address the school nonattendance issue in children and adolescents is understanding their own subjective perceptions [12]. The School Refusal Assessment Scale is one tool which is available for measuring perceptions related to school attendance [13–16]. However, this scale may not always be feasible or practical to use. In Japan, state-level education board offices are also exploring potential variable that may impact students’ perceptions of attending school to support their school attendance. For instance, the Saitama Educational Board has explored a strategy aimed at enhancing positive relationships with peers and school teachers to sustain students’ positive perceptions of school attendance [17]. This strategy is based on concepts and teaching guidelines shared between elementary and junior high schools [17]. Building on a model developed for elementary school students, the board is working to identify a model for junior high school students that also emphasized the importance of positive relationships with friends and school teachers [18]. The model for elementary school students showed that students’ “positive perceptions of attending school” was directly influenced by their “positive perceptions of relationships with friends and school teachers.” Their “positive perceptions of attending school” was directly influenced by their positive perceptions of “current circumstance,” “subjective health status,” and “having people to share experiences and thoughts with,” but not strongly [18]. If the model in junior high school students also proves valid, it could be incorporated into consecutive school-based programs from elementary to junior high school levels, supporting teachers who implement evidence-based school programs. Exploring alternative methods for assessing students ‘perceptions of attending school, such as the strategy developed by the Saitama Educational Board, may be necessary to support school attendance and promote positive relationships between students and teachers.

### Hypothesis development

We aimed to examine our hypothetical model for junior high school students. They are in a period of human development where they experience rapid physical and mental changes. Their thoughts and actions are strongly influenced by peers, and peer attachment security is associated with the ability to form close relationships, which supports positive mental health in adulthood [19]. Adolescents strive for independence from their parents; thus, they spend more time with friends than with parents, and friends become the primary source of interaction and influence [20]. While these relationships can be a source of strength, they can also be a source of stress and conflict [21]. Changes in relationships, meeting new people, and complex schedules as children become older, may lead to their stress and mental health challenges [22]. Junior high school students in Japan are prone to experience particular difficulties in interpersonal relationships and perceive lower self-esteem than their counterparts in Korea and China [23].

Heightened emotional reactivity and issues with emotional regulation can place adolescents at a greater risk of developing generalized anxiety, eating disorders, depression, and social anxiety [24]. Poorer mental health status, with or without diagnoses, may increase the risk for school nonattendance [9]. Key determinants linked to school nonattendance include aggression [16], emotional disorders [25, 26], depression [27], social anxiety [10], and unhappiness at school [28].

Improving help-seeking behaviors and self-support are recommended for adolescents to improve coping and mental health [29, 30]. However, self-seeking support can be difficult to facilitate. For instance, a systematic review revealed that two key reasons that young people do not seek or access help were limited mental health knowledge and perceived social stigma and embarrassment [31]. In one study, nearly 41% of 276 Japanese junior high school students with self-harming issues had never “asked for help” [32].

**Fig. 1:**
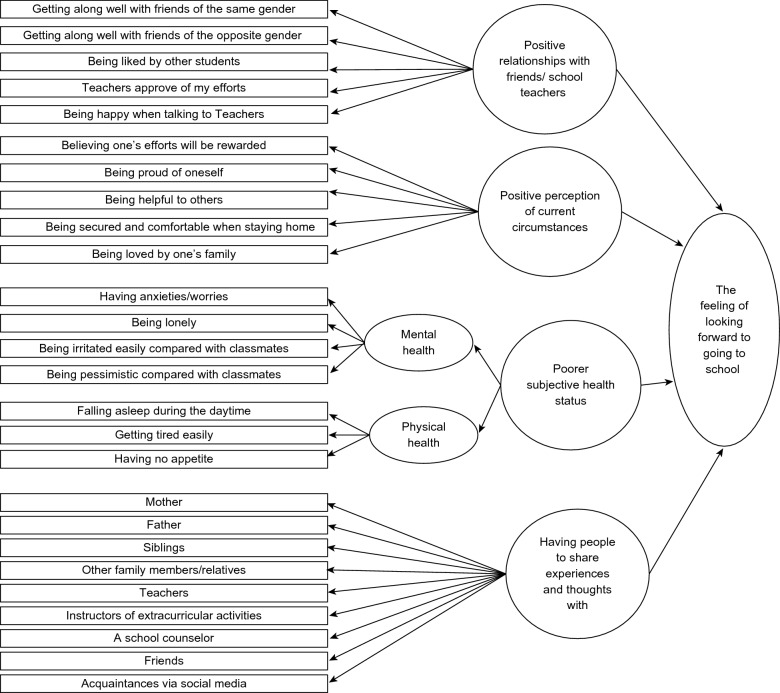
A hypothetical model

We hypothesized that junior high school students’ feelings and perceptions of attending school (the feeling of looking forward to going to school) was directly influenced by their perceptions of relationships with friends and school teachers, current circumstances, subjective health status, and having people to share experiences and thoughts with. Among those variables, we hypothesized that positive perceptions of attending school was positively influenced by the variables of positive perceptions of relationships with friends and school teachers, current circumstances, and having people to share experiences and thoughts with. The inclusion of the latent variable labeled as "perceptions of poor health status" signifies the importance of adhering to the recommended practice of identifying and reporting both physical and mental health conditions and encouraging individuals to actively seek support for them [4]. We hypothesized that positive perceptions of attending school would be negatively influenced by poorer subjective health status. Figure 1 shows our hypothetical model. Ovals represent latent variables (“Positive relationships with friends/school teachers”, “Positive perception of current circumstances,” “Poorer subjective health status (Mental health and Physical health),” and “Having people to share experiences and thoughts with”) and rectangles represent observed variables (each item of questionnaire). The observed variables are described in the questionnaire section below.

## Methods

### Setting

The current study was a cross-sectional study conducted collaboratively between Saitama Prefectural University and the Saitama Educational Board. The educational board manages 415 state-run junior high schools (93% of total junior high schools) in Saitama prefecture (state level), Japan. We focused on students in their 2nd year of junior high school who attended state-run junior high schools in Saitama prefecture according to the educational board survey plan. They were either 13 or 14 years old during the data collection period. There were approximately 59,200 students in the 2nd year of junior high school (approximately 30,300 boys and 28,900 girls). Saitama prefecture, located north of Tokyo, has the third largest residential area used in which housing predominates, and the fourth highest population density out of 47 prefectures in Japan. The Saitama government issued reports comparing the southern, northern, western, and eastern areas based on the population and tax revenue. The Saitama educational board intended to obtain data from 5,900 students in the 2nd year of junior high school, 10% of the total population (approximately 59,000 students in total) due to the additional workload of the board members. The board estimated that the return rate would be 70% based on their previous surveys; thus, the distribution numbers would be approximately 8,400. The board planned to distribute the questionnaire to 2,100 students in each area. School districts in each area were randomly selected by the board to meet the distribution amount. All school district managers and school principals from the area provided permission to conduct the survey. Ninety-three state-run junior high schools were randomly selected from each governmental area group in Saitama.

The original questionnaire described below was distributed between November 2019 and March 2020. Local managers chose the periods for conducting the survey. Prior to the distribution, area managers visited the schools, described the aim of this survey to school teachers, and obtained their agreement to conduct the survey. Data collection was completed by the teachers in their own classrooms.

### Participants

There were 8,945 distributions and 6,429 responses (72% response rate) with a consent to participate in this study. Among the collected data, 184 questionnaires were excluded because of missing data (10% of 19 items), which was 2.9% of the responses. The educational board did not perform imputation for the missing data and wished to capture actual responses from students. A total of 6,245 responses (97% of the collected responses) were used for analysis (Additional file 1: Table S1).

### Questionnaire development procedures

Overall, junior high school students, being older, were expected to understand and respond to a larger number of items with more abstract and complex concepts included than the younger students in a previous study [18]. However, given their experience working in and around schools, the development team strongly believed that a shorter tool was desirable; therefore, brevity and a short list of items remained the target. To examine the model, an original questionnaire, as an extension version, was developed through collaboration between members of the Saitama Educational Board and faculty members of Saitama Prefectural University with a specialty in education. Similar to the previous study [18], several meetings, including face-to-face discussions, videoconferences and email discussions, were held between the members of the development team to refine the items and wording of the questionnaire for the current study.

### Variables

Our hypothetical model had four variables that influenced perceptions toward attending school. Question items were added to other variables based on the previous model for elementary school students [18]. The variables were (1) positive relationships with friends and school teachers, (2) positive perceptions of current circumstances, (3) poorer subjective health status, and (4) the people the students shared their experiences and thoughts with.

### Questionnaire

There were 19 items in the questionnaire. The first item was for capturing students felt about attending school. The item wording was “I am looking forward to going to my school.” The responses were 4 = strongly agree, 3 = agree, 2 = disagree, and 1 = strongly disagree.

To capture positive relationships with friends and school teachers, items asking whether students got along well with friends of the same gender and the opposite gender were considered. The following five items were positively worded: “I get along well with friends of the same gender,” “I get along well with friends of the opposite gender,” “I am liked by other students,” “School teachers approve of my efforts” and “I am happy when talking to school teachers.” For the five items, the responses were 3 = very much, 2 = a little bit, and 1 = not at all.

To capture positive perceptions of current circumstances, previous insights were discussed. For instance, junior high school students in Japan with positive perceptions of current circumstances were more likely to perceive secure feelings [33]. Noncognitive skills [34] are valued for the development of future careers [4]. Junior high school students in Japan experience crucial identity development as well as transitioning between education and further training or work as they being to explore future careers when choosing high schools [4]. State-level educational board offices, including the Saitama Educational Board, foster the ability to believe that effort can help students achieve goals, recognize their strengths, help others, and feel secure and comfortable [17]. The following five items were positively worded: “I believe my efforts will be rewarded,” “I am proud of myself,” “I am helpful to others,” “I am secure and comfortable when staying at home,” and “I am loved by my family.” For the five items, the responses were 3 = very much, 2 = a little bit, and 1 = not at all.

To capture poorer subjective health status, we referred to a guideline for school nurses [4]. The guideline listed symptoms, composed of mental and physical health status, to let students reveal their health concerns such as having anxieties or worries, perceptions of being lonely, poor quality of sleep, tiredness and no appetite. We also referred to a checklist for junior high school students, developed by another state-run educational board [35], to create concrete sentences. In addition, people in a homogeneous society such as Japan are more likely to think and behave like other people in the group do and are more likely to become uncomfortable when they feel differently from others [36]. Therefore, text to show the comparison were added. There were seven items to capture poorer subjective health status. Four items were for mental health and three items were for physical health. Items for mental health status were “I have anxieties and/or worries,” “I am lonely,” “I am irritated easily compared with my classmates” and “I am pessimistic compared with my classmates.” Among them, the responses for the first and second items were 3 = a lot, 2 = a little bit, and 1 = not at all. The responses for the third and fourth items were 3 = very much, 2 = no difference, and 1 = not at all. Items for asking their physical health were “I fall asleep during the daytime,” “I get tired easily” and “I have no appetite.” For the three items, the responses were 3 = frequently, 2 = sometimes, and 1 = not at all.

The 19th item was designed to capture the people the students shared their experiences and thoughts with. Students may have some human resources and choose persons according to topics they wish to discuss. For instance, junior high school students in Japan, Korea, and China solicited advice from teachers, including extracurricular instructors, about their study-related problems, and asked for advice from friends regarding problems or worries about peer and family relations; however, a large number of students had received no formal counseling or support [23]. The following nine options were used: (a) my mother, (b) my father, (c) my siblings, (d) other family members/relatives, (e) school teachers, (f) instructors of extracurricular activities, (g) a school counselor, (h) friends, and (j) acquaintances via social media. The item also asked the participants the frequency of accessing those persons. The responses were 3 = frequently, 2 = sometimes, 1 = rarely, and 0 = there is no such person. Additionally, the students were asked to indicate their age (in years) and sex (male or female).

### Ethics

The research protocol was reviewed and approved by the Research Ethics Committee at Saitama Prefectural University (No. 19078-extension). Consent from students and their parents were obtained.

### Statistical methods

To examine the hypothesized model, we employed a structural equation model (SEM). Before conducting analysis with SEM, we observed descriptive statistics, comparison in responses between male and female students using Cramer’s* V*, and correlations between variables using Spearman’s rank correlation coefficient. The analysis was conducted with HAD 17.0 [37], SPSS v.26 for Japanese (IBM, Japan), and *M*plus version 8.7 [38].

We observed the data characteristics. In societies that value conformity and homogeneity, such as Japan, observing group-level characteristics is rare because state-run schools are guided by a unified education policy. To confirm this, we observed the intraclass correlation coefficient (ICC), which identified group homogeneity. A high ICC results in a biased error variance in conventional regression models, overestimating the relationship between variables. An ICC of 0.25 and higher indicates that much of the variation in the dependent variables is due to the features of groups rather than the characteristics of individuals [39, 40].

We then compared the response characteristics between male and female students according to the items because mental health risks have been reported to be higher in female students than in male students [41]. For the comparisons, we used Cramer’s V, which indicates how strongly two categorical variables are associated, with 1 indicating a strong association and zero indicating no association. Values of 0.1, 0.3, and 0.5 are considered small, medium, and large effect sizes, respectively [42]. Moreover, we observed Spearman’s rank correlation coefficient between the variables. Coefficients were interpreted as limited (0.00–0.25), fair (0.25–0.50), moderate (0.50–0.75) and excellent (0.75–1.0) [43].

Confirmatory factor analysis (CFA) was applied to examine the factorial structure of poorer subjective health status before examining the whole model because it had a second-order structure. In the CFA, no value of the model fit indices was calculated because the standard error of the model parameter estimates was not obtained. We constrained the path coefficient value from poorer subjective health status (a superordinate concept) to the mental health component (a subordinate concept) and to the physical health component (a subordinate concept) to be 1. *M*plus, a software package, provides the root mean square error of approximation (RMSEA), the comparative fit index (CFI) and the Tucker–Lewis index (TLI) to observe model fit indices [44]. The RMSEA is based on the analysis of residuals, with smaller values indicating a better fit to the data [44]. Values below 0.05 indicate a good fit, those between 0.05 and 0.08 indicate a reasonable fit, and those above 0.1 indicate a poor fit [45]. The CFI and TLI are the most commonly used incremental indicators of fit in SEM, and both measure the proportionate improvement in model fit by comparing the hypothesized model in which structure is imposed with the less restricted nested baseline model [45]. CFI and TLI values greater than 0.9 indicate a good model fit [46]. Using those model fit indices, we examined the factorial structure of the latent variable of poorer subjective health status conservatively.

To examine our hypothetical model, we used an SEM with ordinal data and the modified weighted least squares method (WLSMV). We modified the model based on the obtained values of the path coefficients and model fit indices such as the RMSEA, CFI, and TLI. Items with low path coefficient values were excluded when the procedure improved the model fit indices.

## Results

### Participants

Among the 6,245 students, 2,592 (41.51%) were boys, 2,820 (45.16%) were girls, and 833 (13.34%) were students of uncategorized sex. Across the area groups, no significant difference was observed in the percentages between boys and girls (0.029 for Cramer’s *V*, *p* = 0.208). Every student was either 13 or 14 years old.

### Descriptive data

Table 1 shows the values for the valid sample number, interclass correlation, and *p* value for each question item. Across the items, the valid data rates were 98% and higher. No question item showed an ICC value of 0.05 or higher, indicating that all data could be described with individual-level variances and that area-specific characteristics were ignorable.

**Table 1:** Values in valid sample, interclass correlation, and reliability for each question item (n = 6245)

Item wording	Valid sample		ICC		
	n	%		95% CI Lower	95% CI Upper
1. Looking forward to going to my school	6164	98.70	0.019**	0.008	0.067
2. Getting along well with friends of the same gender	6200	99.28	0.001	0.000	0.008
3. Getting along well with friends of the opposite gender	6192	99.15	0.010**	0.004	0.040
4. Being liked by other students	6174	98.86	0.017**	0.007	0.063
5. School teachers approve of my efforts	6182	98.99	0.009**	0.003	0.035
6. Being happy when talking to school teachers	6188	99.09	0.013**	0.005	0.047
7. Believing one’s efforts will be rewarded	6232	99.79	0.005**	0.001	0.022
8. Being proud of oneself	6228	99.73	0.006**	0.002	0.023
9. Being helpful to others	6207	99.39	0.011**	0.004	0.043
10. Being secured and comfortable when staying at home	6227	99.71	0.002	0.000	0.011
11. Being loved by one’s family	6212	99.47	0.003*	0.001	0.016
12. Having anxieties/worries	6223	99.65	0.024**	0.010	0.083
13. Being lonely	6211	99.46	0.010**	0.004	0.038
14. Being irritated easily compared with classmates	6188	99.09	0.005**	0.001	0.020
15. Being pessimistic compared with classmates	6184	99.02	0.002	0.000	0.010
16. Falling asleep during the daytime	6211	99.46	0.003*	0.001	0.015
17. Getting tired easily	6208	99.41	0.005**	0.001	0.020
18. Having no appetite	6207	99.39	0.004**	0.001	0.019
Persons I talk to when sharing my experiences and thoughts with					
a. Mother	6179	98.94	0.015**	0.006	0.055
b. Father	6136	98.25	0.006**	0.002	0.023
c. Siblings	6142	98.35	0.007**	0.002	0.029
d. Other family members/relatives	6172	98.83	0.013**	0.005	0.047
e. School teachers	6187	99.07	0.019**	0.008	0.069
f. Instructors of extracurricular activities	6185	99.04	0.004**	0.001	0.018
g. A school counselor	6162	98.67	0.000	−0.001	0.005
h. Friends	6192	99.15	0.011**	0.004	0.041
j. Acquaintances via social media	6186	99.06	0.002	0.000	0.012

*ICC* interclass correlation, *CI* confidence interval

^*^p < 0.01, **p < 0.001

### Response characteristics

Table 2 shows the response characteristics according to the response alternatives of each item. The distribution of the use of response alternatives indicates a heavy orientation toward “very much” for the item “getting along well with friends of the same gender” (84%) and relatively few observations of “frequently” for the item “a school counselor” (1%). In addition, there was no significant difference in response frequencies between boys and girls across items (Additional file 2: Table S2).

**Table 2:** Response characteristics according to response alternatives in each item

Item	Valid response (n)	Response alternatives (%)			
		Strongly agree	Agree	Disagree	Strongly disagree
1. Looking forward to going to my school	6164	32.15	45.28	16.86	5.71
		Very much	A little bit	Not at all	
2. Getting along well with friends of the same gender	6200	84.44	15.68	1.89	
3. Getting along well with friends of the opposite gender	6192	29.62	44.99	25.39	
4. Being liked by other students	6174	21.72	60.95	17.33	
5. School teachers approve of my efforts	6182	30.25	54.35	15.40	
6. Being happy when talking to school teachers	6188	26.76	48.04	25.19	
7. Believing one’s efforts will be rewarded	6232	65.07	28.82	6.11	
8. Being proud of oneself	6228	23.60	47.59	28.81	
9. Being helpful to others	6207	26.24	53.05	20.70	
10. Being secured and comfortable when staying at home	6227	74.67	20.91	4.42	
11. Being loved by one’s family	6212	59.29	34.35	6.36	
		A lot	A little bit	Not at all	
12. Having anxieties/worries	6223	34.20	42.15	23.65	
13. Being lonely	6211	15.71	31.87	52.71	
		Very much	No difference	Not at all	
14. Being irritated easily compared with classmates	6188	13.61	51.03	35.36	
15. Being pessimistic compared with classmates	6184	13.79	38.29	47.91	
		Frequently	Sometimes	Not at all	
16. Falling asleep during the daytime	6211	16.00	43.60	40.40	
17. Getting tired easily	6208	46.86	42.17	10.97	
18. Having no appetite	6207	18.33	35.07	46.59	
Persons I talk to when sharing my experiences and thoughts with		Frequently	Sometimes	Rarely	No one such person
a. Mother	6179	61.40	28.58	9.06	0.95
b. Father	6136	27.59	38.85	25.73	7.82
c. Siblings	6142	31.11	26.49	30.35	12.05
d. Other family members/relatives	6172	16.67	36.96	41.87	4.50
e. School teachers	6187	14.16	41.05	43.74	1.05
f. Instructors of extracurricular activities	6185	15.10	23.19	37.30	24.41
g. A school counselor	6162	1.04	5.05	57.24	36.68
h. Friends	6192	65.12	24.55	9.56	0.78
j. Acquaintances via social media	6186	3.86	5.85	22.15	68.14

Table 3 shows the values for correlation coefficients between items 1 and 18. No variable showed a significant and positive correlation with the item “I am looking forward to going to school” at an excellent or moderate level. Five significant and positive correlations at a moderate level were observed as follows: between “being liked by other students” and “school teachers approve of my efforts” (*r* = 0.511), between “school teachers approve of my efforts” and “being happy when talking to school teachers” (*r* = 0.552), between “being proud of oneself” and “being helpful to others” (*r* = 0.607), between “being secure and comfortable when staying at home” and “being loved by one’s family” (*r* = 0.507), and between “having anxieties/worries” and “being lonely” (*r* = 0.557). Table 3 also indicates that the correlation coefficients among the items do not exhibit a pattern of values with noticeably high correlations, implying the absence of multicollinearity. Table 4 demonstrates that the correlation coefficients between individuals regarding sharing experiences and thoughts with. No variable showed significant and positive correlations at an excellent or moderate level. This finding further supports the conclusion that multicollinearity does not exist in the model.

**Table 3:** Spearman’s rank correlation coefficient between items 1 and 18

	1	2	3	4	5	6	7	8	9	10	11	12	13	14	15	16	17	18
1	1	0.301*	0.258*	0.345*	0.355*	0.404*	0.383*	0.367*	0.376*	0.224*	0.272*	−0.176*	−0.241*	−0.072*	−0.005	−0.127*	−0.177*	−0.163*
2		1	0.228*	0.330*	0.237*	0.180*	0.220*	0.219*	0.234*	0.169*	0.189*	−0.099*	−0.195*	−0.047*	−0.030	−0.038*	−0.055*	−0.028
3			1	0.303*	0.269*	0.272*	0.136*	0.226*	0.239*	0.070*	0.125*	−0.040*	−0.061*	0.020	0.036*	0.027	−0.015	−0.004*
4				1	0.511*	0.333*	0.267*	0.429*	0.458*	0.219*	0.334*	−0.164*	−0.232*	−0.129*	−0.058*	−0.078*	−0.132*	−0.095*
5					1	0.552*	0.322*	0.435*	0.491*	0.242*	0.387*	−0.107*	−0.177	−0.077*	−0.011*	−0.093*	−0.127*	−0.120*
6						1	0.328*	0.315*	0.364*	0.240*	0.317*	−0.025	−0.088*	−0.038*	0.060*	−0.077*	−0.077*	−0.104*
7							1	0.425*	0.395*	0.333*	0.335*	−0.055*	−0.135*	−0.047*	0.026	−0.072*	−0.094*	−0.085*
8								1	0.609*	0.271*	0.362*	−0.201*	−0.205*	−0.052*	−0.036*	−0.064*	−0.174*	−0.133*
9									1	0.317*	0.459*	−0.135*	−0.184*	−0.084*	−0.028	−0.085*	−0.156*	−0.116*
10										1	0.507*	−0.053*	−0.158*	−0.090*	−0.025	−0.060*	−0.080*	−0.081*
11											1	−0.104*	−0.208*	−0.116*	−0.039*	−0.094*	−0.137*	−0.106*
12												1	0.557*	0.123*	0.145*	0.108*	0.127*	0.061*
13													1	0.140*	0.129*	0.071*	0.096*	0.044*
14														1	0.137*	0.107*	0.181*	0.155*
15															1	0.133*	0.235*	0.141*
16																1	0.318*	0.171*
17																	1	0.235*
18																		1

1: Looking forward to going to my school, 2: Getting along well with friends of the same gender, 3: Getting along well with friends of the opposite gender, 4: Being liked by other students, 5: School teachers approve of my efforts, 6: Being happy when talking to school teachers, 7: Believing one’s efforts will be rewarded, 8: Being proud of oneself, 9: Being helpful to others, 10. Being secured and comfortable when staying at home, 11: Being loved by one’s family, 12: Having anxieties/worries, 13: Being lonely, 14: Being irritated easily compared with classmates, 15: Being pessimistic compared with classmates, 16; Falling asleep during the daytime, 17: Getting tired easily, 18: Having no appetite

^*^p < 0.01

**Table 4:** Spearman’s rank correlation coefficient between people the students shared experiences and thoughts with

	a	b	c	d	e	f	g	h	j
a. Mother	1	0.439*	0.367*	0.346*	0.329*	0.161*	−0.095*	0.340*	−0.187*
b. Father		1	0.369*	0.357*	0.329*	0.210*	−0.051*	0.252*	−0.128*
c. Siblings			1	0.343*	0.380*	0.266*	−0.039*	0.306*	−0.094*
d. Other family members/relatives				1	0.322*	0.174*	−0.040*	0.287*	−0.079*
e. School teachers					1	0.317*	−0.050*	0.365*	−0.075*
f. Instructors of extracurricular activities						1	0.190*	0.219*	0.027*
g. A school counselor							1	−0.095*	0.215*
h. Friends								1	−0.188*
j. Acquaintances via social media									1

^*^p < 0.01

### Examining the second-order structure

Table 5 shows the results of a CFA approach for the structure of subjective health status (all *p* < 0.001). The model fit indices were 0.106 for RMSEA (90% CI: 0.100, 0.111), 0.907 for CFI, and 0.850 for TLI. The path coefficient value from the latent variable to the mental health component was 0.840, and the value from the latent variable to the physical health component was 0.585. The factor loading values were between 0.882 and 0.335. We believe that the two items with lower factor loading values (below 0.4) accounted for the poor RMSEA and TLI values. These two items with low path coefficients were expected to be excluded when examining the model.

**Table 5:** Results of confirmatory factor analysis for subjective health status

Item/correlation	Factor loadings		
	Estimate	SE	Estimate/SE
12. Having anxieties/worries	0.882*	0.013	62.119
13. Being lonely	0.828*	0.014	60.596
14. Being irritated easily compared with classmates	0.338*	0.015	22.290
15. Being pessimistic compared with classmates	0.335*	0.015	21.788
16. Falling asleep during the daytime	0.485*	0.017	28.413
17. Getting tired easily	0.832*	0.021	39.622
18. Having no appetite	0.423*	0.017	24.606
Path coefficient values from subjective health status to the two components			
Mental health component (items 12–15)	0.840	0.029	29.271
Physical health component (items 16–18)	0.585	0.019	31.004

*SE* Standardized Error

^*^p < 0.001

### Initial model examination

The initial model examination using SEM obtained the follows values: 7285.871 for the chi-square test of model fit (314 degrees of freedom, *p* < 0.001), 0.060 for RMSEA (90% CI 0.058, 0.061), 0.909 for CFI, and 0.899 for TLI. The model showed that “positive perceptions of attending school” variable was directly and positively impacted by “positive relationships with friends and school teachers” (0.422 for the path coefficient value), “current circumstances” (0.113 for the path coefficient value), and “having people to share experiences and thoughts with” (0.047 for the path coefficient value) variables (all *p* < 0.001). The model also showed that “positive perceptions of attending school” variable was directly and negatively impacted by “poorer subjective health status” variable (–0.217 for the path coefficient value, *p* < 0.001). The path coefficient value from poorer subjective health status to the mental health component was 0.752, and the path coefficient value to the physical health component was 0.656 (both *p* < 0.001), supporting the second-order structure.

The path coefficient values between latent variables were similar to the values obtained in the model modification; thus, values were addressed in the results for the model modification. The path coefficient values from the latent variables to items were 0.944 and 0.373 (all *p* < 0.001). There were three items with lower path coefficient values: “being pessimistic compared with classmates” (0.255, *p* < 0.001) from the mental health component in the poorer subjective health status as well as “a school counselor” (−0.035, *p* = 0.030) and “acquaintances via social media” (−0.261, *p* < 0.001) from having people to share experiences and thoughts with. These three items were excluded when the model was modified.

### Model modification

**Fig. 2:**
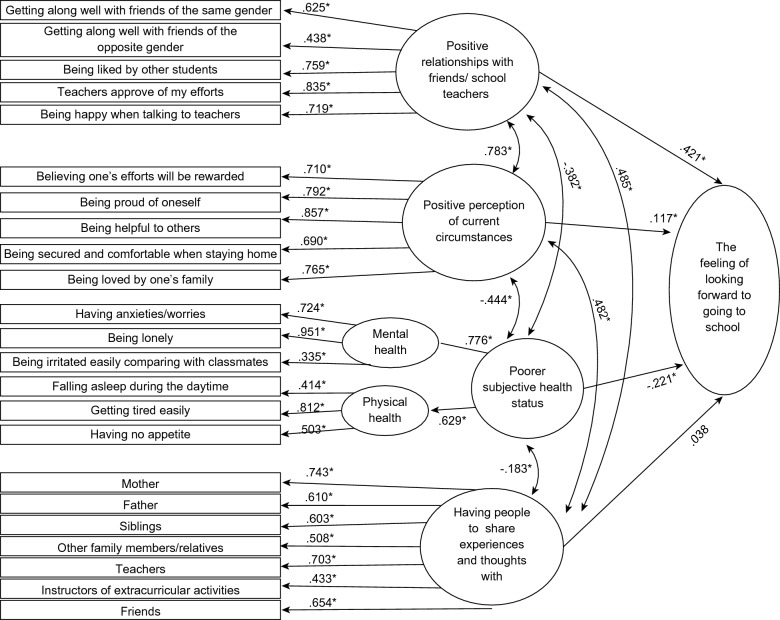
Modified model using SEM. Numbers are path coefficients, *p < 0.001

Figure 2 shows the modified model using SEM. The chi-square test of model fit was 73279.024 (degrees of freedom was 276, *p* < 0.001). Model fit indices were improved from the initial examination as follows: 0.055 for RMSEA (90% CI 0.054, 0.057), 0.937 for CFI, and 0.928 for TLI. The model showed that “positive perceptions of attending school” variable was directly impacted by “positive perceptions of relationships with friends and school teachers” (0.421 for the path coefficient value) and “positive perceptions of current circumstances” (0.117 for the path coefficient value) variables (both *p* < 0.001). “Positive perceptions of attending school” variable was directly and negatively impacted by “poorer subjective health status” variable (–0.221 for the path coefficient value, p < 0.001). The path coefficient value from “poorer subjective health status” to “mental health” component was 0.776, and the path coefficient value to “physical health” component was 0.629 (both *p* < 0.001), supporting the second-order structure. “Having people to share experiences and thoughts with” variable (0.038 for the path coefficient value, *p* = 0.016) was not found to be significantly related to “positive perceptions of attending school”.

Among the latent variables, “positive perceptions of relationships with friends and school teachers,” “current circumstances,” and “having people to share experiences and thoughts with” correlated significantly and positively with each other, showing the path coefficient values between 0.783 and 0.482 (all *p* < 0.001). “Poorer subjective health status” was significantly and negatively correlated with the three latent variables above, showing path coefficient values between –0.444 and –0.183 (all p < 0.001).

For “positive perceptions of relationships with friends and school teachers,” the path coefficient values for the items were between 0.835 and 0.438 (all *p* < 0.001). For “positive perceptions of current circumstances,” the path coefficient values for the items were between 0.857 and 0.690 (all *p* < 0.001). For “poorer subjective health status,” the path coefficient values for the items were between 0.951 and 0.335 for “mental health” component and between 0.812 and 0.414 for “physical health” component (all *p* < 0.001). For “having people to share experiences and thoughts with,” the path coefficient values for the variables were between 0.743 and 0.433 (all *p* < 0.001). The item “being irritated easily compared with classmates” showed a value below 0.4 for the path coefficient; however, excluding the item did not improve the values for the model fit indices.

## Discussions

In this study, we identified the structural relations among positive perceptions of attending school by junior high school students in state-run schools in Saitama, Japan. The model showed that students’ “positive perceptions of attending school” was positively and directly influenced by their perceptions of “positive relationships with friends and school teachers.” The model also showed that students’ “positive perceptions of attending school” was negatively and directly influenced by their “perceptions of poorer subjective health status.” Students with positive perceptions of attending school were more likely to perceive their relationships with friends and school teachers positively. Students who lacked positive perceptions of attending school were more likely to perceive poorer subjective health status.. In addition, the identified model did not support a strong connection between students’ perceptions of attending school and their perceptions of current circumstances as well as between the students’ perceptions of attending school and having people to share experiences and thoughts with. Students’ perceptions of relationships with friends and school teachers, current circumstances, and having people to share experiences and thoughts with were positively correlated with each other. These three latent variables were negatively correlated with poorer subjective health status.

In the comparison between the current model for junior high school students and the previous model for elementary school students [18], three common characteristics were observed. First, “students’ perceptions of attending school” was directly influenced by their perceptions of “relationships with friends and school teachers” and “subjective health status.” Second, “perceptions of attending school” was not strongly influenced by their perceptions of “current circumstances” and “having people to share experiences and thoughts with.” Third, the path coefficient values for school teachers from the latent variable of “having people to share experiences and thoughts with” were higher than those for father, siblings, and other family members or relatives.

The second-order structure for the latent variable of subjective health status was supported for junior higher school students. However, no such second-order structure was used among elementary school students. For capturing junior high school students’ physical and mental changes, the second-order structure was important. It is important to note that the variables that were used to create the latent variables in this study may need to be improved in future studies.

The findings of the current study are important for several reasons. It is crucial for students and policymakers to prioritize the subjective health and well-being of junior high school students and provide them with support and resources to navigate this critical period of their lives. While the Saitama Educational Board has implemented programs to establish positive relationships between peers and teachers and improve help-seeking behaviors [17], junior high school students are still less studied and not as well served by service providers or policymakers compared to younger children [47]. Moreover, the national policy, “Healthy Parents and Children 21,” focuses on newborns, toddlers, and their mothers, despite the fact that suicide has been a major cause of death among adolescents in Japan [48, 49]. Recognizing this issue, the Japanese government has established a new agency to support the dignity of children and reduce the suicide rate in adolescents [50]. Schools can play an important role in promoting health education, reducing stigma, raising awareness, and identifying and supporting adolescents who experience poorer subjective health status [51–54]. In both the current study and the previous study [18], students identified school teaches as individuals with whom they felt comfortable sharing their experiences and thoughts with. Thus, school teachers can play an essential role in understanding and accepting their students. To facilitate this, we recommend that Japanese schools implement the evidence-based questionnaire we developed in their programs to better support their students.

### Clinical implication

The model presented highlights the importance of supporting students in developing and maintaining positive relationships with both friends and teachers, as this significantly influences their overall perception of attending school. This finding may encourage teachers in Japanese state-run schools to adopt new practices that foster such relationships. Additionally, while the impact was not found to be as strong, it is still important to support students in perceiving their current circumstances positively and provide opportunities for them to share their experiences and thoughts with others. Given that students are undergoing rapid physical and mental changes, it is important to focus on subjective health status, and provide resources and support for students who may be struggling with their mental or physical health.

### Strengths and limitations of this study

A large random sample collected through collaboration with the educational board in Saitama, Japan, enabled us to robustly examine our hypothesized model. An excellent response rate was facilitated by the school district managers and school teachers who distributed and collected the original questionnaire. However, this study has a few limitations to consider. First, it was conducted in Japan, which is known for being a homogeneous society. The definition and circumstances surrounding school nonattendance in Japan may differ from those in other countries. As a result, the model's applicability might be limited in more culturally diverse nations or countries with distinct patterns or definitions of school nonattendance. Second, some latent variables to describe students’ feelings and perceptions of attending school may be missing. Because we developed a hypothetical model and an original questionnaire based on the literature, and teacher guidelines used in Japan. The current study was the first attempt to examine the questionnaire in junior high school students. Further examinations may be needed to reveal variables that have the potential to directly and strongly influence issues of school attendance. Third, we did not obtain information regarding whether students had a school nonattendance history and whether they had received support for attendance. Thus, we were unable to compare responses between students with poor versus good attendance. Fourth, we examined students attending state-run junior high schools and in a specific age range. Additionally, behaviors, attitudes, peer pressure and socioeconomic deprivation in junior high school students may be different between cultures [55]. The model must be examined in different settings, areas and age groups.

## Conclusions

This study explored the perspective of 6245 junior high school students in Japan regarding school attendance and related factors. The study found that students’ positive perceptions of school attendance were directly and positively influenced by their positive perceptions of relationships with friends and school teachers. Poorer subjective health status was found to have a direct and negative impact on their positive perceptions of school attendance. The findings of this study have important implications for school teachers and district managers who wish to promote positive school attendance among students. Specifically, the study suggests that programs aimed at developing positive relationships with friends and school teachers may be particularly effective in promoting positive perceptions of school attendance among students.

## Supplementary Information

**Additional file 1: Table S1.** Demographic characteristics according to area groups.

**Additional file 2: Table S2.** Comparisons of responses between boys and girls according to response alternatives in each item.

## Data Availability

The data are stored at the Saitama Educational Board office and restricted for research use only before April 2023. The data are not publicly available. Please contact the corresponding author to discuss data access.

## Abbreviations

CFA: Confirmatory factor analysis
CFI: Comparative fit index
ICC: Intraclass correlation coefficient
RMSEA: Root mean square error of approximation
SEM: Structural equation model
TLI: Tucker–Lewis index
WLSMV: Weighted least squares method
