# Supplementary material for: Factors influencing junior high school students’ perceptions of attending school in Japan
Source: Child Adolesc Psychiatry Ment Health. 2023 Jul 4;17:86. doi: 10.1186/s13034-023-00631-w (PMC10320959; doi:10.1186/s13034-023-00631-w)
Supplement: Supplementary file 1 — Additional file 1: Table S1. Demographic characteristics according to area groups. [file 13034_2023_631_MOESM1_ESM.docx]

| **Additional file**  **Table 1** Sample characteristics according to area groups | | | | | |
| --- | --- | --- | --- | --- | --- |
|  | Total | Area | | | |
|  |  | North | South | East | West |
| Distribution number | 8945 | 1980 | 2358 | 2122 | 2485 |
| Collection number | 6429 | 1679 | 1470 | 1496 | 1784 |
| Collection rate (%) | 71.9 | 84.8 | 62.3 | 70.5 | 71.8 |
| Analyzed number | 6245 | 1632 | 1427 | 1453 | 1733 |
| Analyzed/collected (%) | 97.1 | 97.2 | 97.1 | 97.1 | 97.1 |
