# Supplementary material for: Factors influencing junior high school students’ perceptions of attending school in Japan
Source: Child Adolesc Psychiatry Ment Health. 2023 Jul 4;17:86. doi: 10.1186/s13034-023-00631-w (PMC10320959; doi:10.1186/s13034-023-00631-w)
Supplement: Supplementary file 2 — Additional file 2: Table S2. Comparisons of responses between boys and girls according to response alternatives in each item. [file 13034_2023_631_MOESM2_ESM.docx]

| **Additional FIle**  **Table 2** Comparisons of responses between male and female according to response alternatives in each item | | | | | | |
| --- | --- | --- | --- | --- | --- | --- |
| Item and response alternatives | | | Frequency (%) | | | *V* |
|  |  | | Total | Male | Female |  |
| 1. Looking forward to going to my school | | |  |  |  | .034 |
|  | Strongly agree | | 32.15 | 30.88 | 33.39 |  |
|  | Agree | | 45.28 | 46.72 | 43.81 |  |
|  | Disagree | | 16.86 | 17.04 | 16.79 |  |
|  | Strongly disagree | | 5.71 | 5.36 | 6.01 |  |
| 2. Getting along well with friends of the same gender | | |  |  |  | .034 |
|  | Very much | | 84.44 | 85.15 | 83.40 |  |
|  | A little bit | | 13.68 | 12.75 | 14.90 |  |
|  | Not at all | | 1.89 | 2.11 | 1.71 |  |
| 3. Getting along well with friends of the opposite gender | | |  |  |  | .021 |
|  | Very much | | 29.62 | 28.47 | 30.13 |  |
|  | A little bit | | 44.99 | 46.45 | 44.55 |  |
|  | Not at all | | 25.39 | 25.08 | 25.32 |  |
| 4. Being liked by other students | | |  |  |  | .033 |
|  | Very much | | 21.72 | 23.50 | 20.76 |  |
|  | A little bit | | 60.95 | 59.48 | 61.79 |  |
|  | Not at all | | 17.33 | 17.01 | 17.44 |  |
| 5. School teachers approve of my efforts | | |  |  |  | .022 |
|  | Very much | | 30.25 | 31.15 | 29.34 |  |
|  | A little bit | | 54.35 | 54.37 | 55.10 |  |
|  | Not at all | | 15.40 | 14.48 | 15.56 |  |
| 6. Being happy when talking to school teachers | | |  |  |  | .044* |
|  | Very much | | 26.76 | 24.93 | 28.81 |  |
|  | A little bit | | 48.04 | 48.93 | 47.02 |  |
|  | Not at all | | 25.19 | 26.14 | 24.17 |  |
| 7. Believing one’s efforts will be rewarded | | |  |  |  | .012 |
|  | Very much | | 65.07 | 64.62 | 65.73 |  |
|  | A little bit | | 28.82 | 29.00 | 28.13 |  |
|  | Not at all | | 6.11 | 6.38 | 6.14 |  |
| 8. Being proud of oneself | | |  |  |  |  |
|  | Very much | | 23.60 | 29.27 | 18.17 | .152** |
|  | A little bit | | 47.59 | 47.60 | 48.04 |  |
|  | Not at all | | 28.81 | 23.12 | 33.78 |  |
| 9. Being helpful to others | | |  |  |  | .062** |
|  | Very much | | 26.24 | 28.73 | 23.67 |  |
|  | A little bit | | 53.05 | 52.41 | 54.29 |  |
|  | Not at all | | 20.70 | 18.87 | 22.04 |  |
| 10. Being secured and comfortable when staying home | | |  |  |  | .034 |
|  | Very much | | 74.67 | 73.64 | 76.13 |  |
|  | A little bit | | 20.91 | 22.18 | 19.43 |  |
|  | Not at all | | 4.42 | 4.18 | 4.44 |  |
| 11. Being loved by one’s family | | |  |  |  | .046* |
|  | Very much | | 59.29 | 57.83 | 61.20 |  |
|  | A little bit | | 34.35 | 36.43 | 32.20 |  |
|  | Not at all | | 6.36 | 5.74 | 6.60 |  |
| 12. Having anxieties/worries | | |  |  |  | .126** |
|  | Having a lot | | 34.20 | 29.28 | 38.34 |  |
|  | Having a little bit | | 42.15 | 42.10 | 42.64 |  |
|  | Not at all | | 23.65 | 28.62 | 19.01 |  |
| 13. Being lonely | | |  |  |  | .086** |
|  | Frequently | | 15.71 | 13.54 | 17.59 |  |
|  | Sometimes | | 31.57 | 28.74 | 33.08 |  |
|  | Not at all | | 52.71 | 57.72 | 49.32 |  |
| 14. Being irritated easily compared with classmates | | |  |  |  | .028 |
|  | Very much | | 13.61 | 12.48 | 14.38 |  |
|  | No difference | | 51.03 | 51.73 | 50.34 |  |
|  | Not at all | | 35.36 | 35.79 | 35.28 |  |
| 15. Being pessimistic compared with classmates | | |  |  |  | .185** |
|  | Very much | | 13.79 | 7.49 | 19.85 |  |
|  | No difference | | 38.29 | 38.98 | 38.88 |  |
|  | Not at all | | 47.65 | 53.53 | 42.27 |  |
| 16. Falling asleep during the daytime | | |  |  |  | .035 |
|  | Frequently | | 16.00 | 14.31 | 16.69 |  |
|  | Sometimes | | 43.60 | 43.41 | 43.37 |  |
|  | Not at all | | 40.40 | 42.28 | 39.94 |  |
| 17. Getting tired easily | | |  |  |  | .073** |
|  | Frequently | | 46.86 | 43.05 | 49.84 |  |
|  | Sometimes | | 42.17 | 44.53 | 40.68 |  |
|  | Not at all | | 10.97 | 12.42 | 9.48 |  |
| 18. Having no appetite | | |  |  |  | .023 |
|  | Frequently | | 18.33 | 16.66 | 18.43 |  |
|  | Sometimes | | 35.07 | 35.88 | 34.97 |  |
|  | Not at all | | 46.59 | 47.46 | 46.60 |  |
| Persons I talk to when sharing my experiences and thoughts with | | |  |  |  |  |
|  | a. Mother | |  |  |  | .183** |
|  |  | Frequently | 61.40 | 52.44 | 69.35 |  |
|  |  | Sometimes | 28.58 | 34.57 | 23.45 |  |
|  |  | Rarely | 9.06 | 12.28 | 6.06 |  |
|  |  | No one such person | 0.95 | 0.70 | 1.14 |  |
|  | b. Father | |  |  |  | .092** |
|  |  | Frequently | 27.59 | 30.88 | 23.63 |  |
|  |  | Sometimes | 38.85 | 39.11 | 39.58 |  |
|  |  | Rarely | 25.73 | 23.36 | 28.04 |  |
|  |  | No one such person | 7.82 | 6.66 | 8.75 |  |
|  | c. Siblings | |  |  |  | .104** |
|  |  | Frequently | 31.11 | 26.99 | 34.52 |  |
|  |  | Sometimes | 26.49 | 26.35 | 26.13 |  |
|  |  | Rarely | 30.35 | 34.93 | 26.41 |  |
|  |  | No one such person | 12.05 | 11.73 | 12.94 |  |
|  | d. Other family members/relatives | |  |  |  | .046* |
|  |  | Frequently | 16.67 | 16.56 | 16.17 |  |
|  |  | Sometimes | 36.96 | 35.26 | 38.15 |  |
|  |  | Rarely | 41.87 | 42.90 | 42.04 |  |
|  |  | No one such person | 4.50 | 5.28 | 3.64 |  |
|  | e. School teachers | |  |  |  | .040 |
|  |  | Frequently | 14.16 | 14.04 | 13.47 |  |
|  |  | Sometimes | 41.05 | 41.38 | 40.16 |  |
|  |  | Rarely | 43.74 | 43.33 | 45.76 |  |
|  |  | No one such person | 1.05 | 1.25 | 0.61 |  |
|  | f. Instructors of extracurricular activities | |  |  |  | .026 |
|  |  | Frequently | 15.10 | 14.57 | 14.17 |  |
|  |  | Sometimes | 23.19 | 23.87 | 22.29 |  |
|  |  | Rarely | 37.30 | 37.70 | 37.81 |  |
|  |  | No one such person | 24.41 | 23.87 | 25.74 |  |
|  | g. A school counselor | |  |  |  | .067** |
|  |  | Frequently | 1.04 | 0.86 | 1.11 |  |
|  |  | Sometimes | 5.05 | 5.77 | 3.96 |  |
|  |  | Rarely | 57.24 | 54.65 | 60.37 |  |
|  |  | No one such person | 36.68 | 38.72 | 34.56 |  |
|  | h. Friends | |  |  |  | .136** |
|  |  | Frequently | 65.12 | 58.64 | 70.13 |  |
|  |  | Sometimes | 24.55 | 27.47 | 22.61 |  |
|  |  | Rarely | 9.56 | 12.68 | 6.91 |  |
|  |  | No one such person | 0.78 | 1.21 | 0.36 |  |
|  | j. Acquaintances via social media | |  |  |  | .063** |
|  |  | Frequently | 3.86 | 2.97 | 4.20 |  |
|  |  | Sometimes | 5.85 | 5.63 | 5.94 |  |
|  |  | Rarely | 22.15 | 24.18 | 19.54 |  |
|  |  | No one such person | 68.14 | 67.23 | 70.32 |  |
| *V*: Cramer’s V  **p* < .01, ***p* < .001 | | | | | | |
